# Socioeconomic gap between neighborhoods of Budapest: Striking impact on stroke and possible explanations

**DOI:** 10.1371/journal.pone.0212519

**Published:** 2019-02-20

**Authors:** Ildikó Szőcs, Dániel Bereczki, András Ajtay, Ferenc Oberfrank, Ildikó Vastagh

**Affiliations:** 1 Semmelweis University, Department of Neurology, Budapest, Hungary; 2 Institute of Experimental Medicine of the Hungarian Academy of Sciences, Budapest, Hungary; Montana State University, UNITED STATES

## Abstract

**Introduction:**

Hungary has a single payer health insurance system offering free healthcare for acute cerebrovascular disorders. Within the capital, Budapest, however there are considerable microregional socioeconomic differences. We hypothesized that socioeconomic deprivation reflects in less favorable stroke characteristics despite universal access to care.

**Methods:**

From the database of the National Health Insurance Fund, we identified 4779 patients hospitalized between 2002 and 2007 for acute cerebrovascular disease (hereafter ACV, i.e. ischemic stroke, intracerebral hemorrhage, or transient ischemia), among residents of the poorest (District 8, n = 2618) and the wealthiest (District 12, n = 2161) neighborhoods of Budapest. Follow-up was until March 2013.

**Results:**

Mean age at onset of ACV was 70±12 and 74±12 years for District 8 and 12 (p<0.01). Age-standardized incidence was higher in District 8 than in District 12 (680/100,000/year versus 518/100,000/year for ACV and 486/100,000/year versus 259/100,000/year for ischemic stroke). Age-standardized mortality of ACV overall and of ischemic stroke specifically was 157/100,000/year versus 100/100,000/year and 122/100,000/year versus 75/100,000/year for District 8 and 12. Long-term case fatality (at 1,5, and 10 years) for ACV and for ischemic stroke was higher in younger District 8 residents (41–70 years of age at the index event) compared to D12 residents of the same age. This gap between the districts increased with the length of follow-up. Of the risk diseases the prevalence of hypertension and diabetes was higher in District 8 than in District 12 (75% versus 66%, p<0.001; and 26% versus 16%, p<0.001).

**Discussion:**

Despite universal healthcare coverage, the disadvantaged district has higher ACV incidence and mortality than the wealthier neighborhood. This difference affects primarily the younger age groups. Long-term follow-up data suggest that inequity in institutional rehabilitation and home-care should be investigated and improved in disadvantaged neighborhoods.

## Introduction

In the last decades there is a decreasing, but still significant difference in stroke mortality among European countries. The high stroke mortality observed in Eastern Europe increased until the end of the eighties when it began to decrease [1]. In spite of this downward trend, it is still lagging behind Western European countries by a decade [2]. The reason for this gap between East and West is quite complex, but might be dramatically simplified to socioeconomic disparities [3].

To understand the impact of socioeconomic differences on stroke outcome, we need to address three aspects: the exact stroke measures possibly influenced by the socioeconomic status (SES), the size of the population studied, and the intermediating factors that may transfer the effect of SES on outcome.

The results are contradictory regarding to which stroke parameter is the most sensitive to SES. People of lower SES were found to show higher stroke incidence and mortality, higher severity in the acute phase, higher readmission rate to hospital for any vascular event after stroke and to lose more disability-adjusted life-years [4–7]. A report from a European country with a single payer health insurance system, Italy, showed higher incidence and more severe state in the acute phase of stroke but not higher 30-day or 1-year case fatality in the less wealthy subgroups of the population [8].

As to the size of the population studied, Kondo et al have recently suggested that income inequalities of populations larger than 800,000 inhabitants (internationally or within a country) show association between income and poor health. They have also argued that this relationship is more difficult to detect among smaller populations [9]. Nevertheless, other studies report striking differences on a smaller perspective, namely between different social groups within one single country [10].

The intermediating factors by which social status influences stroke measures are little known. Some studies postulate that lifestyle risk factors are responsible for explaining the impact of poverty on stroke outcome [11]. A meta-analysis performed by Kerr et al states that the increased risk of stroke associated with low SES is only partly explained by the known classic vascular risk factors [12]. Disparities in clinical, behavioral risk factors or even in differential access to healthcare facilities were also shown not to explain fully the regional differences in stroke incidence and outcome [11–13]. Brown et al found that even in models adjusted for demographic characteristics, stroke type, behavioral and biologic risk factors, mortality hazard 1 year after stroke was still significantly higher among residents of neighborhoods with the lowest SES compared to those in the highest SES neighborhoods [10]. As a summary of the published data, one might say that intermediating factors are not only little known, but they have not been unequivocally identified yet. Provenance from a socially disadvantaged neighborhood could be an independent risk factor with direct effect on stroke.

Hungary has a single payer health insurance system which makes access to care universal and offers a potential for uniform quality of disease-management, stroke care being completely free of charge. On the other hand, there are important regional differences within the capital, Budapest, regarding the SES of the population of the different districts. Despite our expectations for the single system providing universal healthcare, our workgroup has already identified a tendency for worse outcome in stroke patients of the least wealthy district of Budapest compared to those coming from a wealthier neighborhood [14]. Another comparison of the same districts of Budapest showed that male stroke patients from the lower income district die 12 years younger than their more fortunate fellows residing in the wealthier neighborhood [15]. The two studies cited [14,15] comprised of relatively small number of cases—a few hundred for both—and short follow-up periods.

We aimed to extend the evaluation of the impact of intraurban socioeconomic disparities on outcome to a substantial number of ACV cases of the two neighborhoods performing a long-term follow-up. In order to be able to influence this association in the future, we also aimed to evaluate the possible intermediating factors between socioeconomic disadvantage and ACV.

We consider that we have reached this aim by demonstrating the significant difference between ACV incidence and outcome between the poor and the wealthy neighborhoods, and furthermore by analyzing the associated risk diseases in these districts and the trends of fatality observed at long-term follow-up.

## Material and methods

### Study setting

Hungary has one single payer health insurance system with virtually all in- and outpatient data centralized in the National Health Insurance Fund’s database. Budapest is the capital city of Hungary, with a population of 1.8 million inhabitants living in 23 districts. Out of these, District 8 ranks among the last, while District 12 ranks the 1st in the net annual income list for 2011 [16]. The ranking order on this list changes each year, but these two districts figure invariably on the two extremes of the range. There are no obvious differences between the two districts regarding sex ratio, ethnicity, mother tongue, education, religion, the number of general practitioners or of hospital beds related to the number of inhabitants. Nevertheless, several parameters, all mirroring the general economic well-being of the residents, differ with the advantage of District 12: mean annual taxable income, ratio of ground-floor buildings per block of flats, population density, unemployment (Table 1). Interestingly, age distribution of the population also differs, which is an important fact when we study stroke data, as older age is associated with worse outcome after stroke. Residents of District 12 above 40 years of age represent a higher ratio than those of District 8, and this difference between the two districts increases with advancing age (Table 1). We have considered only patients older than 40 years to have sample groups large enough for statistical analysis for each decade. In order to calculate age standardized rates for the incidence and mortality in the 2 districts, data from the census of Hungary in 2011 and the European Standard Population of 2013 was used [17,18].

**Table 1:** Demographic and socioeconomic features of the two neighborhoods.

Data of census 2011	District 8	District 12
Population	76,250	57,709
Sex ratio (women, %)	53,5%	55%
Education (ratio of college degree)	38.9%	38.6%
No. of inhabitants per general practitioner	1228	1096
Ethnic Hungarian	77%	85%
Domestic non-Hungarian ethnicities together	7.3%	4.9%
Ratio of people aged > 40 years	47%	55%
Ratio of people aged > 70 years	11%	18%
Income (mean annual taxable income, thousand Hungarian Forints)	2,115	3,525
Population density (inhabitants/1 km^2^)	11,131	2,164
Unemployed	8%	3.3%
Ground-floor building / block of flat (ratio)	20.8/100	104/100

### Study design

We have done a retrospective observational cohort study comparing the data of District 8 residents admitted for a new-onset ACV in any hospital of Hungary between 2002 and 2007 with the data of their fellows residing in District 12.

The study has been approved by the Ethics Committee of Semmelweis University, Budapest, Hungary (registration number: 88/2015). As this study was retrospective and non-interventional, performed by accessing the anonymized patient records from the national database of the health insurance fund, there was no need for informed consent from individual patients. All data were fully anonymized at accessing them and before the analysis began. No individual data is published in this manuscript.

First we have identified from the national database of the health insurance fund all patients older than 18 years of age, residing in District 8 or 12 (based on the postal code of their permanent address), hospitalized in Hungary for ACV between the 1^st^ of January 2002 and 31^th^ of December 2007. We have collected data for ACV including ischemic stroke, intracerebral hemorrhage and transient ischemic attack (ICD-10—international classification of diseases 10—I63, I61 or G45). To consider only the acute cases, we have ruled out those patients who were admitted to any hospital with the diagnosis of ischemic or hemorrhagic stroke in the previous 2 years. We have also excluded those without a valid social identification number. We have collected data regarding the associated diseases from the hospital reports. Of these we separately analyzed cardiovascular and metabolic comorbidities that are considered to be stroke risk factors.

As the next step, using record linkage by tracking the patient on an individual basis with help of their encrypted social identification number from the database of the National Health Insurance Fund, we have followed up these patients for hospital readmissions, any recurrent cerebrovascular events, newly established secondary diagnoses, or case fatality until March 2013 (S1 Table). Thus, the follow-up period ranged from 5.2 years (December 2007 to March 2013) to 12.2 years (January 2002 to March 2013).

### Measures of acute cerebrovascular disease considered in the study

Even though neither of the districts studied has more than 80,000 inhabitants, we give the numbers of crude incidence and age-standardized incidence and mortality related to 100,000 inhabitants to be able to compare it to published national or international data. We calculated crude case incidence as the number of index events divided by total person-years during inclusion period (2002–2007).

As to fatality, we do not have data regarding the place and exact cause of death. We have taken into consideration deaths of any cause, not necessarily the direct consequences of ACV. We present fatality data at 30 days, and at 1, 5, and 10 years’ follow-up, as these might add to our knowledge about the difference between the two districts, even if long-term fatality is not strictly attributable to the ACV. First, we analyzed acute cerebrovascular disease overall, then we separately evaluated the largest subgroup of ACV, i.e. ischemic stroke.

### Statistical analysis

Normality of continuous variables was evaluated by the Shapiro-Wilk test. Due to non-normal distribution, age at the index event was compared between the districts by the Mann-Whitney test. As to incidence and mortality z-scores were calculated to compare proportions between the populations of the two districts. Acute and long-term case fatality were compared between the 2 districts by chi-squared test. When comparing multiple age-groups we did not make corrections for multiple comparisons. Direct standardization was used to calculate age-standardized incidence and mortality, using the 2013 European Standard Population for standardization [18]. Kaplan-Meier curves were constructed and the log rank test was used to compare the survival distribution between patients of the 2 districts. The association between mean age at the index stroke and average annual income of the 23 districts was evaluated by Spearman correlation. Statistical significance was set at p <0.05. Statistica for Windows v. 12 (StatSoft, Tulsa, OK) was used for data analysis.

## Results

### General data

We have found 4886 ACV patients altogether, their age ranging from 18 to 104 years. After excluding patients 40 years or younger, 4779 remained (98% of the initial number). Among these patients, 58% were females, 2618 resided in District 8 and 2161 in District 12, respectively. Of all ACV patients, 63% had ischemic stroke, 5% had intracerebral hemorrhage and 32% had transient ischemic attacks (hereafter TIA) with no difference between the two districts. During the follow-up period (until March 2013), 57% of patients passed away.

The minimum period of follow-up exceeded 5 years, so at 5 years we have the follow-up data of all 4779 patients. The patients included at the end of the 2002–2007 baseline period have a lower follow-up time until March 2013. Thus, 10-year follow-up data are available for 3129 patients.

### Incidence of acute cerebrovascular disease and of ischemic stroke

The crude incidence of the indexed ACV was somewhat lower in District 8 (572/100,000/year) than in District 12 (623/100,000/year, Table 2). The age standardized incidence rates for hospitalized ACV cases were higher, though, in the poorer district: 680/100,000/year for District 8 and 518/100,000/year for District 12 (Table 2). Considering only ischemic stroke, the crude incidence and the age-standardized incidence values were all higher for residents of District 8 (Table 2).

**Table 2:** Incidence of ACV and ischemic stroke in the two neighborhoods.

Study data	District 8	District 12	Significance (2-sided p values for z-score)
Crude incidence of ACV	572/100,000/year	623/100,000/year	p<0.01
Age-standardized incidence of ACV	680/100,000/year	518/100,000/year	ns
Crude incidence of ischemic stroke	408/100,000/year	325/100,000/year	p<0.001
Age-standardized incidence of ischemic stroke	486/100,000/year	259/100,000/year	p<0.001

### Age at onset

**Fig 1:**
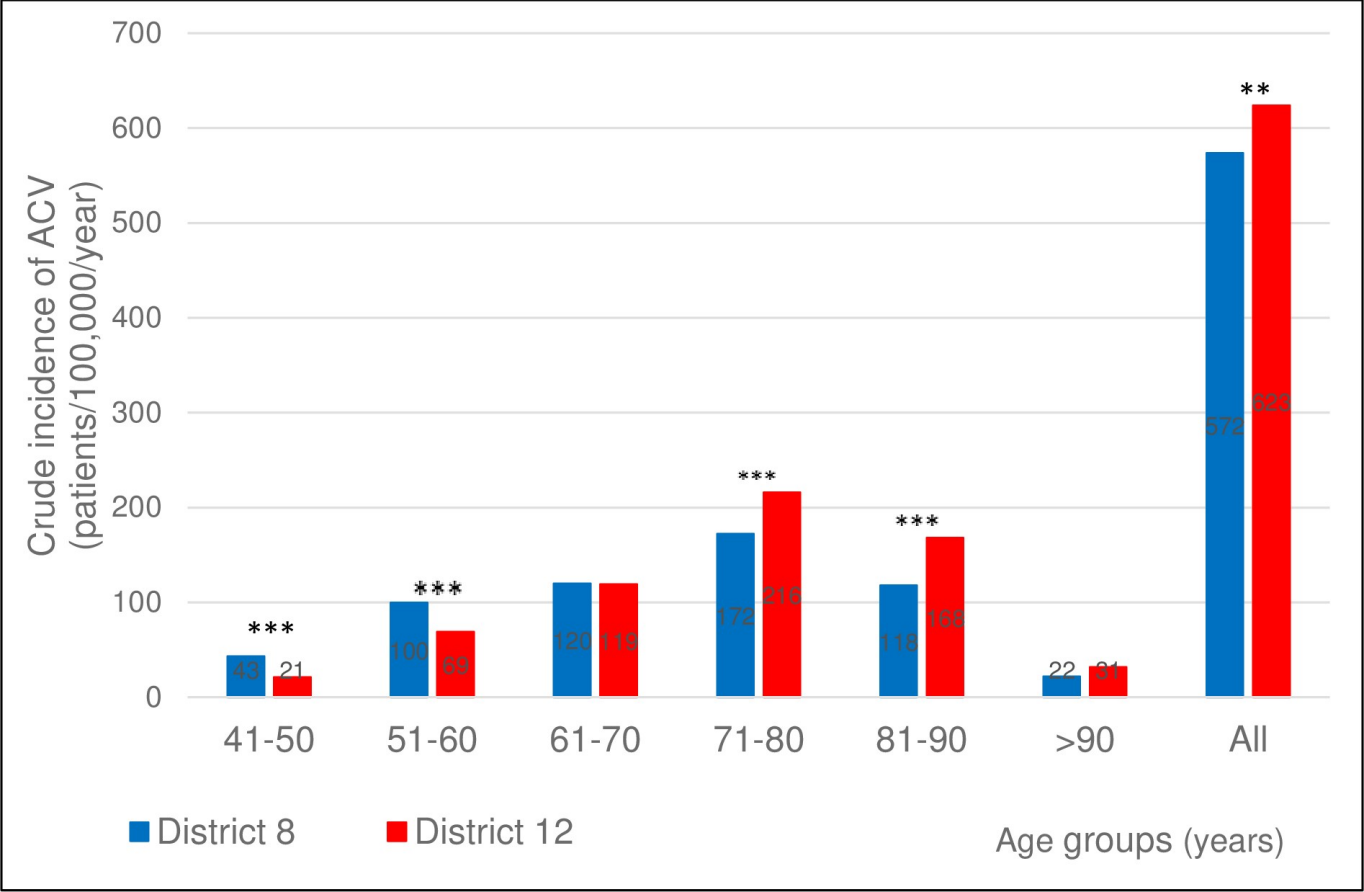
Crude incidence of ACV in the two districts in the different age groups. Crude incidence of ACV (ischemic stroke, intracerebral hemorrhage, and transient ischemia) in the two districts. 2-sided p values for z-score: **p<0.01, ***p<0.001.

The crude incidence of ACV in the 5^th^ and 6^th^ decades of life at the index event was significantly higher for District 8 than for District 12, while the opposite was true for the 8^th^ and 9^th^ decades (Fig 1).

**Fig 2:**
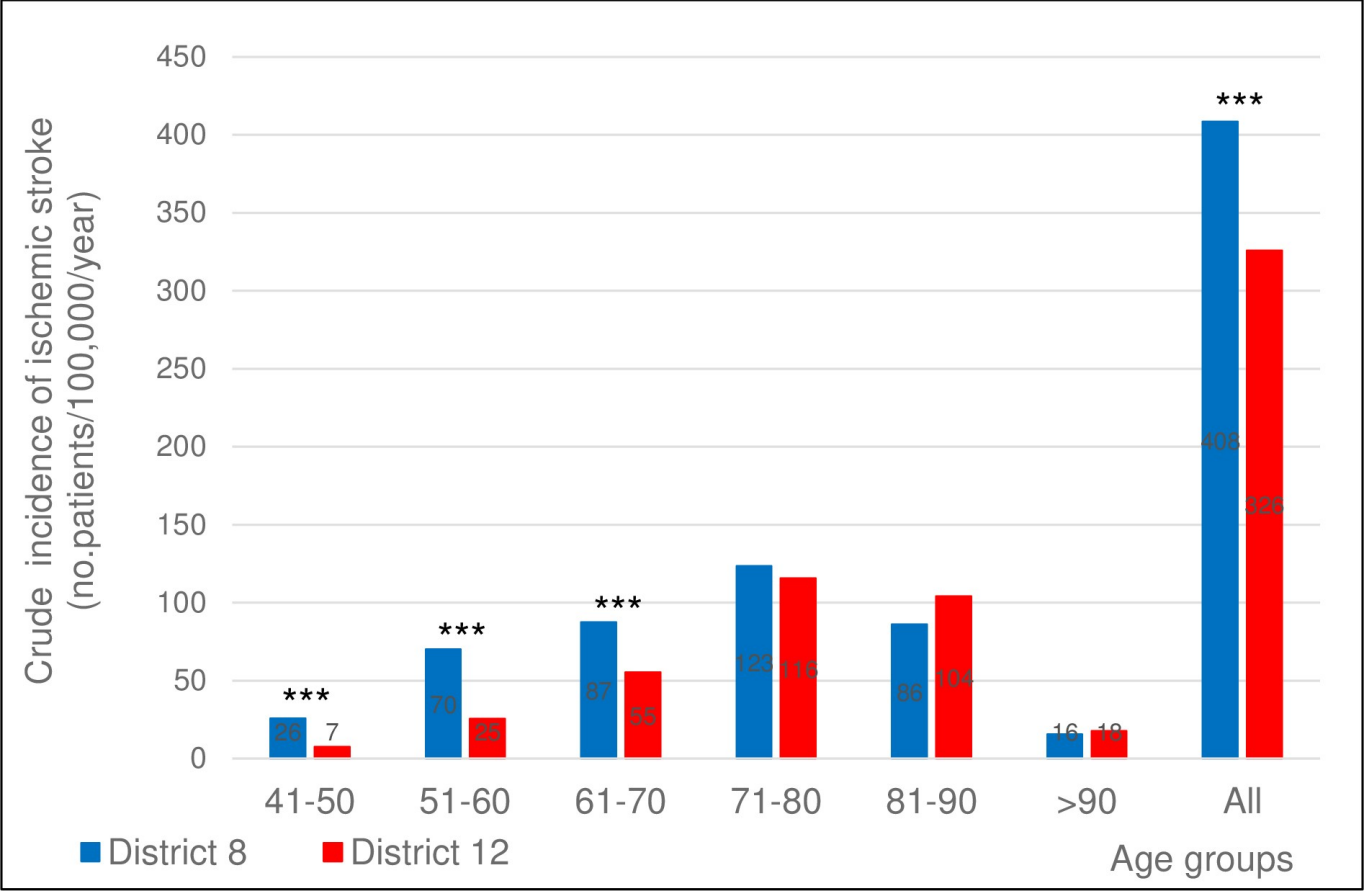
Crude incidence of ischemic stroke in the two districts in the different age groups. Crude incidence of ischemic stroke in the two districts in the different decades of age. 2-sided p values for z-score: ***p<0.001.

As to age at the index ischemic stroke, we have found a significantly higher crude incidence among residents of District 8 compared to District 12 for the 5^th^, 6^th^ and 7^th^ decades of life, with no significant difference for the older age groups (Fig 2).

**Fig 3:**
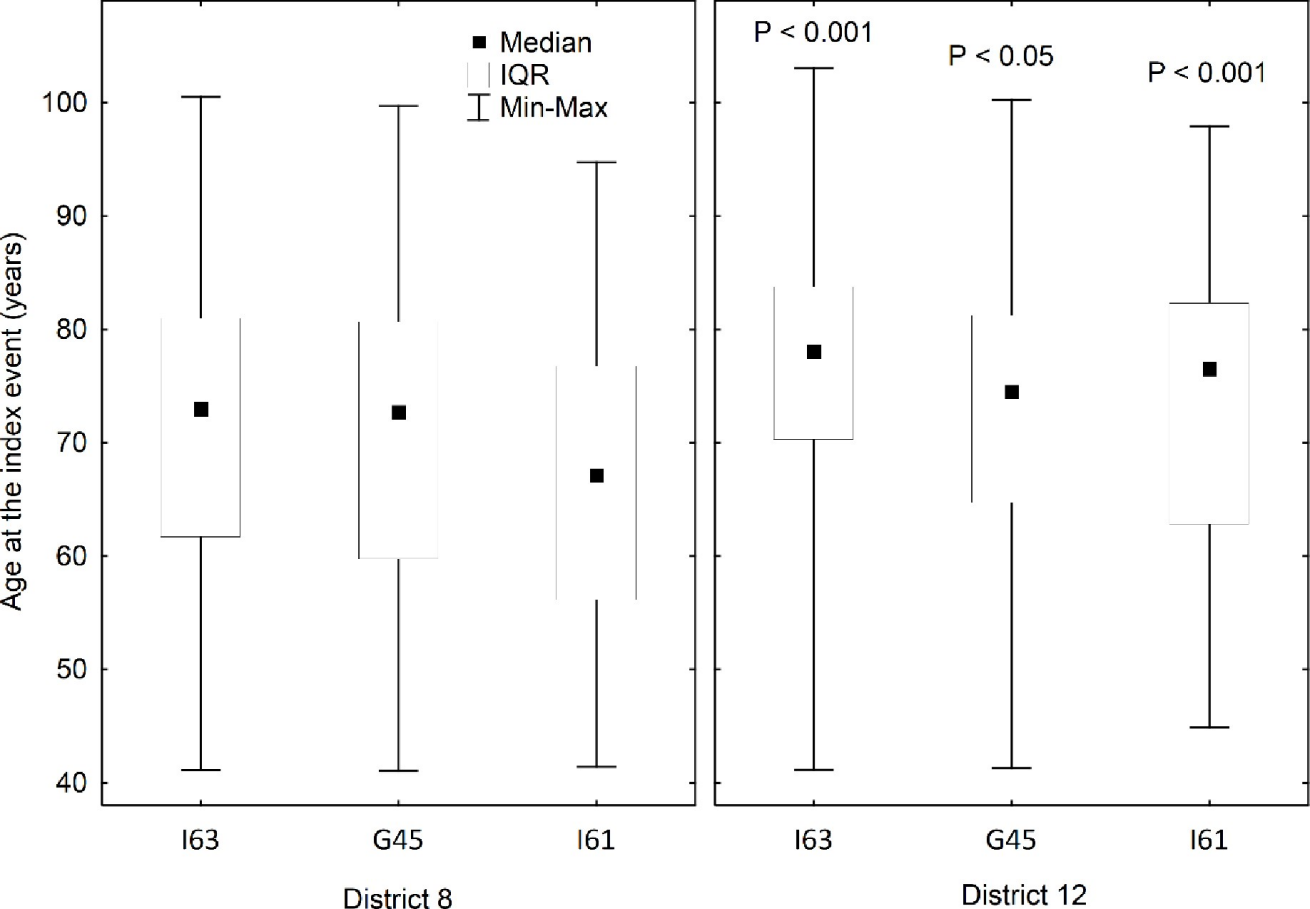
Age at onset of ischemic stroke, intracerebral hemorrhage and transient ischemia in the two districts. Age at onset of ischemic stroke, intracerebral hemorrhage and transient ischemia in the two districts. G45 = transient ischemia, I63 = ischemic stroke, I61 = intracerebral hemorrhage. Statistical significance was calculated with Mann-Whitney test for each diagnostic category between the districts.

Further analyzing the age at disease onset, we saw a similar pattern for all subtypes of ACV: the mean age at onset of TIA, ischemic stroke or intracerebral hemorrhage is significantly lower for District 8 than for District 12 (Fig 3). The ischemic stroke patients are 5 years younger at onset in District 8, the intracerebral hemorrhage cases by 7 years, the TIA cases by 2 years compared to District 12 residents (p<0.05 for all).

**Fig 4:**
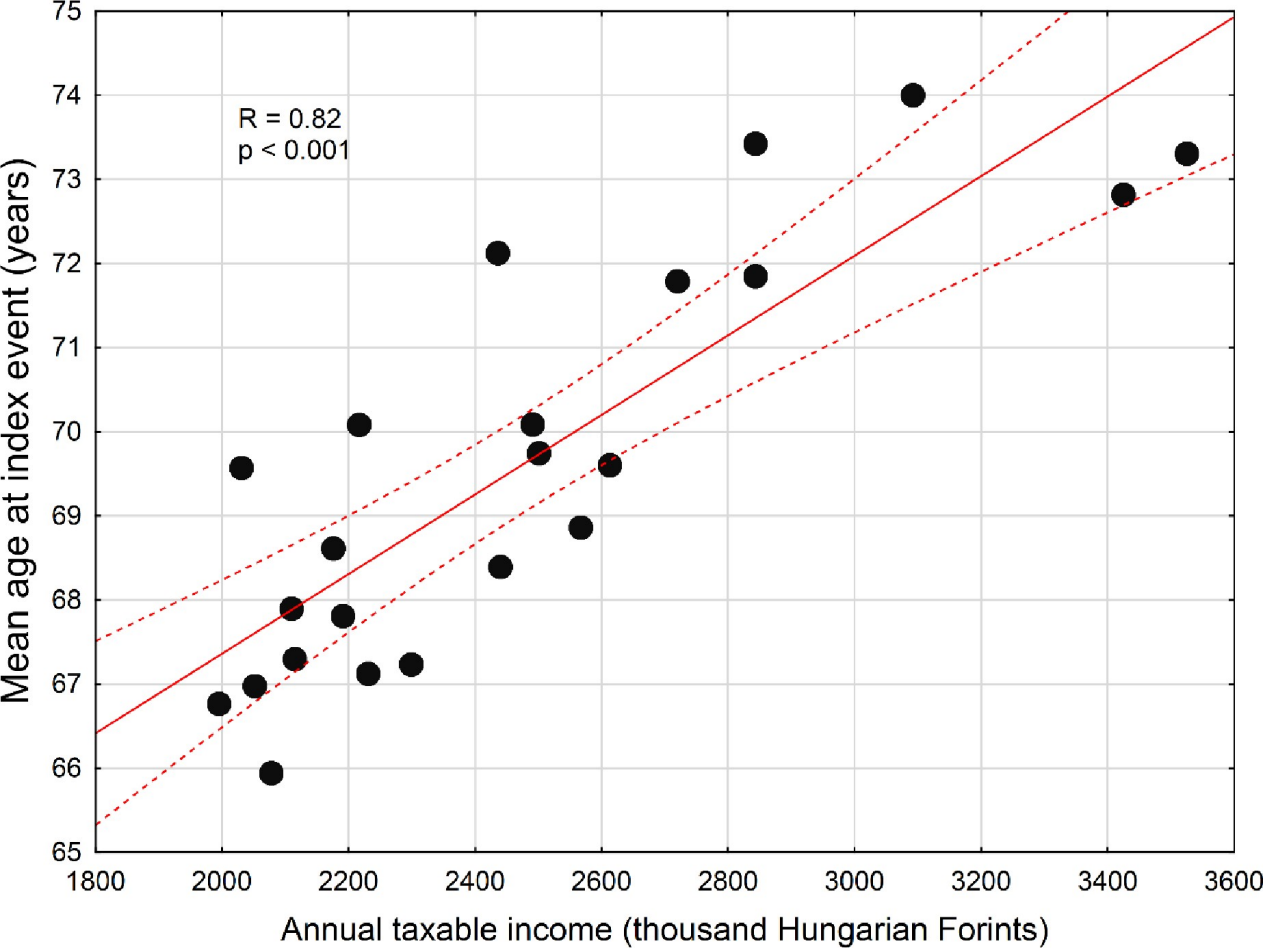
Association of the mean age at index ischemic stroke with annual taxable income of the 23 districts of Budapest. Relationship of the mean age at ischemic stroke with annual taxable income of the 23 districts of Budapest. Each point represents the mean values for one district. Spearman R = 0.82, p < 0.001.

As we found a considerable difference between the two districts of Budapest regarding the age at the index event, we decided to further test our study hypothesis in a supplementary analysis. For this reason, we requested and accessed data of patients with ischemic stroke for all 23 districts of Budapest from an ongoing nationwide project [19] and related the mean age of the patients at the index event to the mean annual income of the respective district for all 23 districts. We have found that the higher the mean annual taxable income, the older the mean age of patients at the index ischemic stroke (Fig 4, p<0.001).

### Outcome of acute cerebrovascular disease and ischemic stroke

#### Case fatality

**Case fatality of ACV** patients with onset between 2002–2007, followed-up until March 2013 showed a difference between the two neighborhoods. Taking into consideration all subtypes together, we saw that case-fatality at 30-days and at 1 year did not differ between the two districts. In contrast, at 5-years follow-up, fatality was significantly higher for District 8 than for District 12 (Table 3).

**Table 3:** Case fatality and mortality of ACV and ischemic stroke in the two neighborhoods.

Study data	District 8	District 12	p value
30 days fatality of ACV	11.5%	11.6%	ns
1 year fatality of ACV	22.5%	21.2%	ns
5 years fatality of ACV	45%	41%	p<0.01
10 years fatality of ACV	86%	85.8%	ns
30 days fatality of ischemic stroke	12.2%	17%	p<0.001
1 year fatality of ischemic stroke	24.6%	30.9%	p<0.001
5 years fatality of ischemic stroke	49%	53%	p<0.05
10 years fatality of ischemic stroke	88%	87%	ns
Crude mortality of ACV	340/100.000/year	338/100.000/year	ns
Age-standardized mortality of ACV	157/100.000/year	100/100.000/year	p<0.001
Crude mortality of ischemic stroke	259/100.000/year	217/100.000/year	p<0.01
Age-standardized mortality of ischemic stroke	122/100000/year	75/100000/year	p<0.001

**Fig 5:**
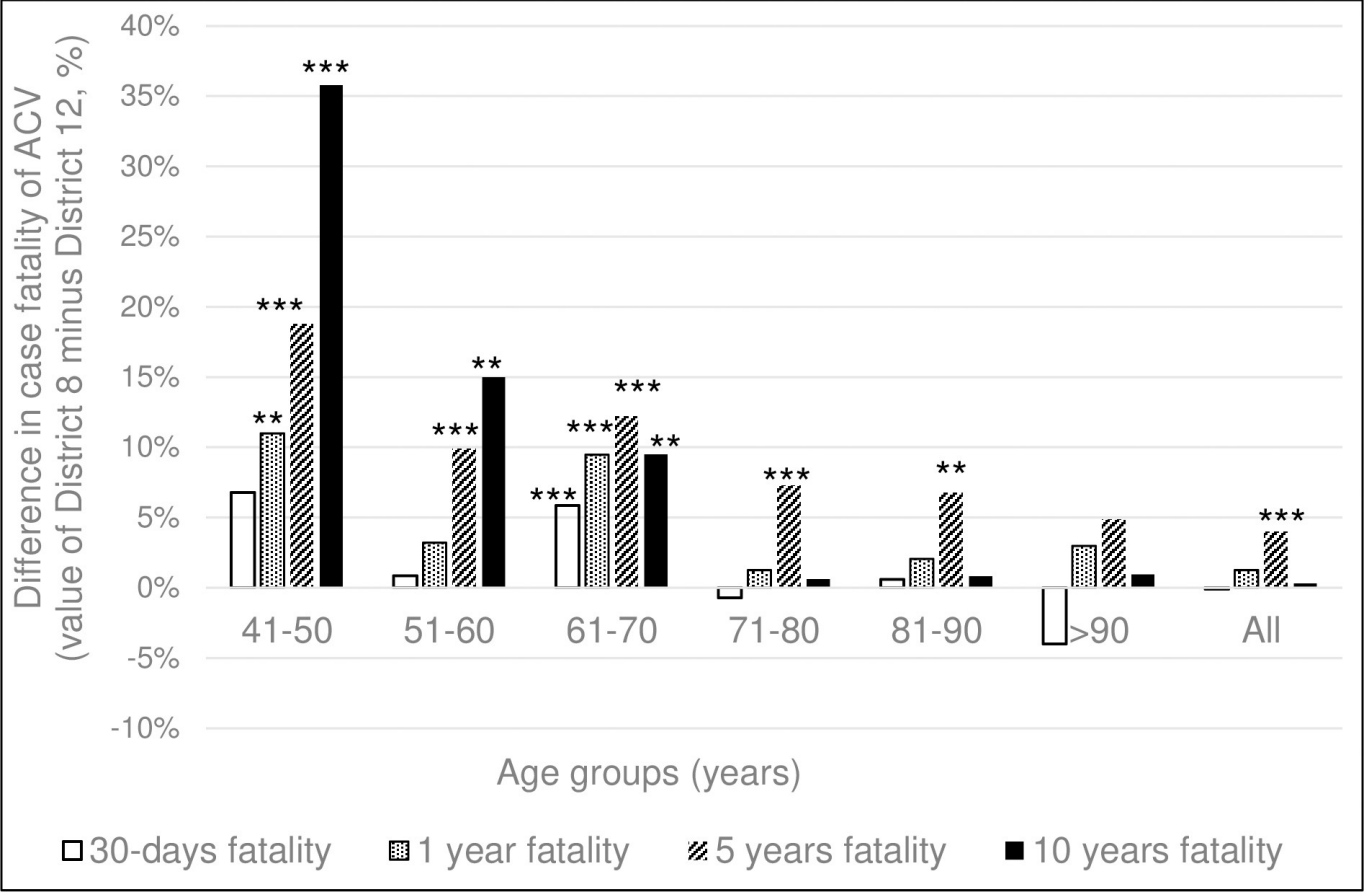
The difference by which the fatality of acute cerebrovascular disease patients of District 8 exceeds that of District 12 in the different age-groups. The difference by which case fatality of District 8 ACV patients exceeds that of District 12 (value of District 8 minus the value of District 12) at 30 days, 1, 5 and 10 years in function of decades of age. Chi-square test. *p<0.05, **p<0.01, ***p<0.001.

Evaluating cases by age at onset, acute case fatality (at 30 days) of ACV patients was significantly higher in District 8 in the 5^th^ and 7^th^ decades (p<0.05 and p<0.01, respectively), with no significant difference in the other age groups (Fig 5.). At long-term follow-up (1, 5 and 10 years), the difference by which the fatality of District 8 ACV patients exceeded that of District 12, increased, especially for the 5^th^, 6^th^, 7^th^ decades (for significance levels see Fig 5).

As to **case fatality of ischemic stroke,** without further dividing them to different age-groups, 30 days and 1-year fatality was significantly higher for the group of District 12 than of District 8. This difference for the disadvantage of District 12 seemed to lessen, but was still significant by 5 years and vanished at 10-years follow-up (Table 3).

**Fig 6:**
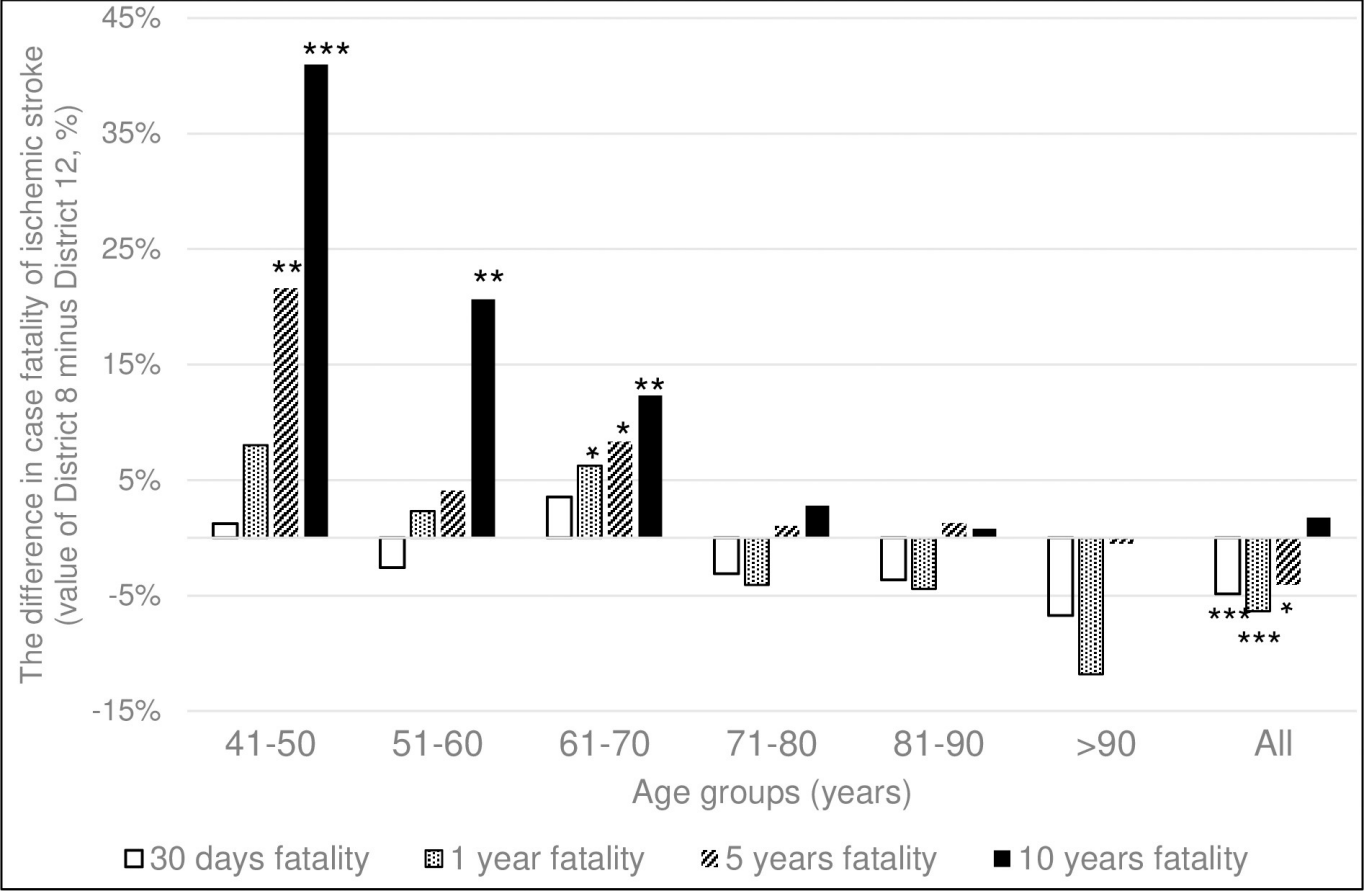
The difference of case fatality of ischemic stroke between the two districts in the separate age-groups. The difference of case fatality of District 8 versus District 12 ischemic stroke patients (value of District 8 minus the value of District 12) at 30 days, 1, 5 and 10 years in function of decades of age. Chi-square test, * p<0.05, **p<0.01, ***p<0.001.

From the results already presented regarding age at the index event, we know that the mean age of District 12 patients was higher compared to that of District 8 for each subtype of ACV. As case fatality of ischemic stroke is knowingly influenced by the age of the patients, we made a sub-analysis. Taking into consideration the age at the index ischemic stroke, we found that case fatality of the younger (5^th^, 6^th^, 7^th^ decades) and the older (above 70 years) patients behaved differently in these two districts. In the groups younger at the event, acute and long-term case fatalities were all higher in the poor District 8 compared to similar groups of the wealthy District 12 (Fig 6), while in the groups with more advanced age at onset, fatality was somewhat, but not significantly higher in District 12 compared to similar groups of District 8 (Fig 6).

#### Mortality

The crude mortality of ACV patients was estimated to 340/100,000/year for District 8 and 338/100,000/year for District 12. The crude mortality of ischemic stroke was 259/100,000/year for District 8 and 217/100,000/year for District 12, respectively (Table 3). The age-standardized values of mortality of ACV and ischemic stroke both showed a larger difference than the crude mortality, with markedly worse results for District 8 (Table 3).

#### Survival

**Fig 7:**
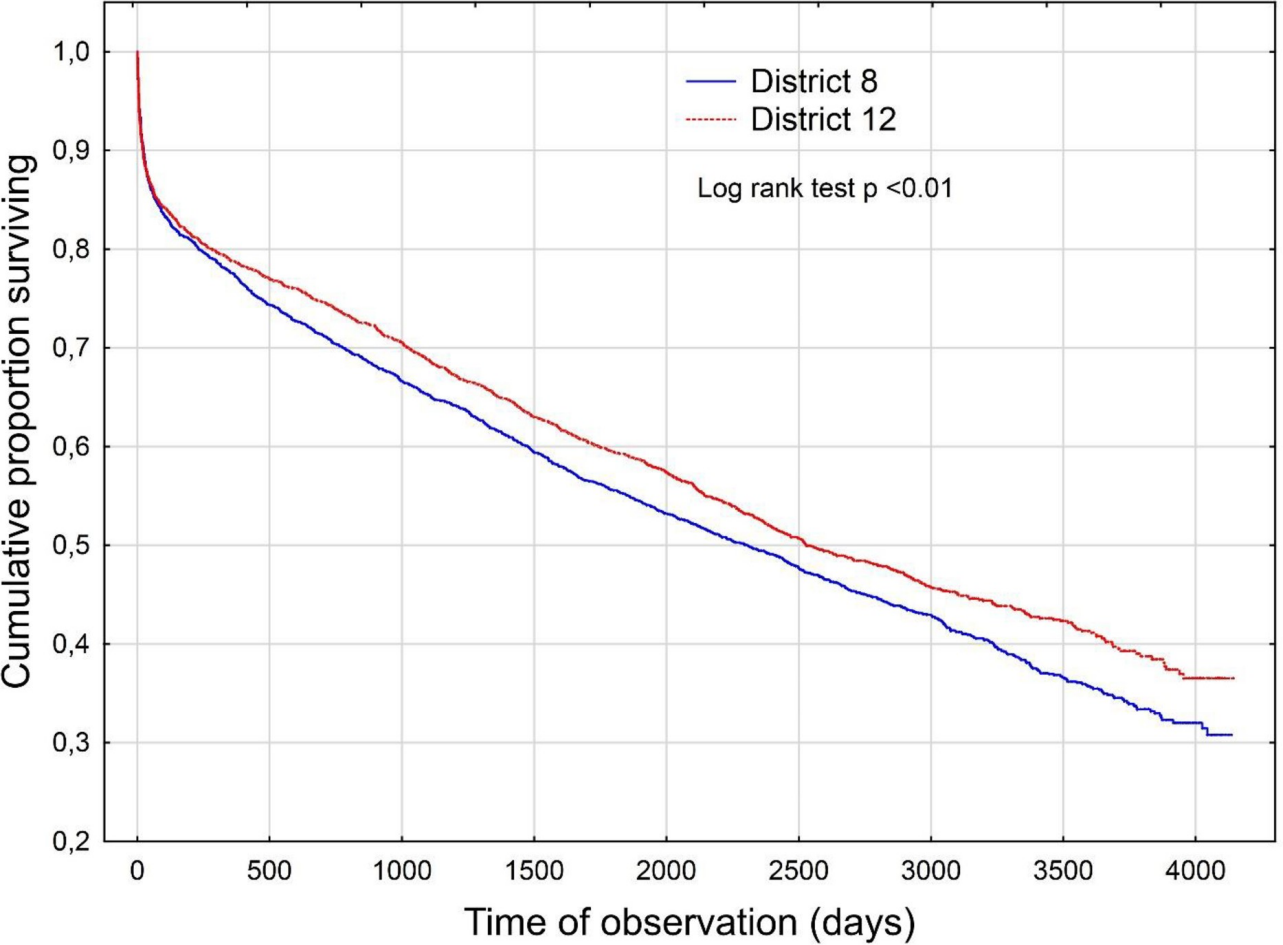
Kaplan-Meier survival curve. Kaplan-Meier survival curve for acute cerebrovascular event cases. Log-rank test, p<0.01.

Having in view the length of survival of all cases from the onset of ACV (followed up from the onset until the end of the follow-up period, i.e. March 2013), we have found that the cumulative proportion of patients surviving was higher in District 12 at each time-point of the follow-up (Fig 7, p<0.01).

### Comorbidities

**Fig 8:**
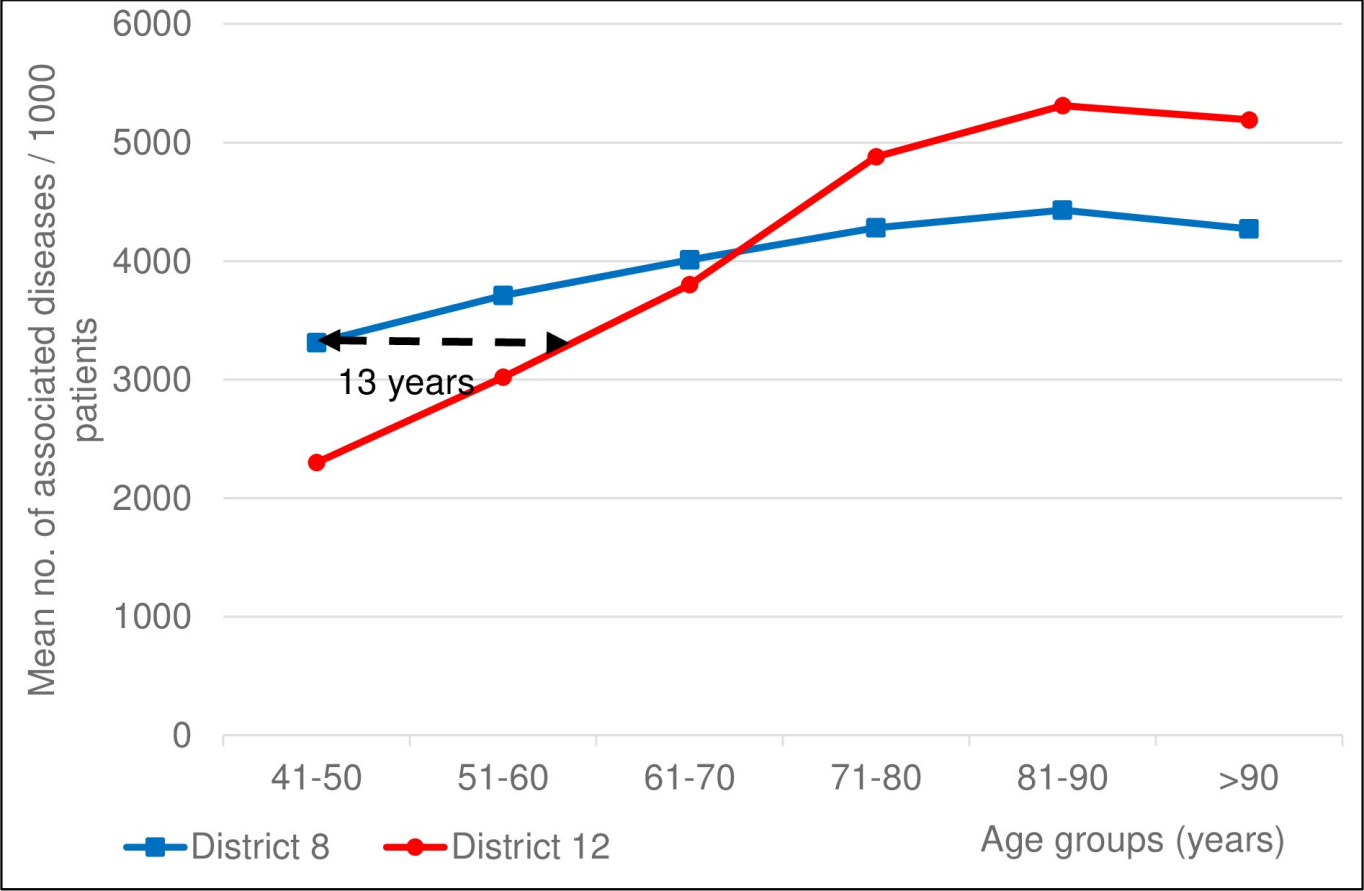
Number of any associated diseases in ischemic stroke patients in 10-year age groups. Mean number of any associated diagnoses per 1000 ischemic stroke patients of the two districts in 10-year age groups. Patients dwelling in District 8 being 41–50 years of age at ischemic stroke onset carry a disease burden as high as their 13 years’ older fellows from District 12.

Regarding comorbidities, we report the data restricted to patients with ischemic stroke, the largest subgroup within ACV. We have analyzed all the ICD-10 codes reported at the hospital discharge and considered them as diseases associated to ischemic stroke. We have noticed that the overall disease burden (i.e. the number of any associated diseases per patient averaged for the respective age group) was significantly higher in younger groups of District 8 patients compared to District 12, this difference vanishing in the age-groups above 70 years (Fig 8). Patients dwelling in District 8 being 41–50 years of age at the index ischemic stroke show a disease burden as high as their 13 years’ older fellows from District 12 based on linear interpolation. In the age groups above 70 years, the trends will be inversed: elderly patients from District 12 show higher frequency of comorbidities.

**Fig 9:**
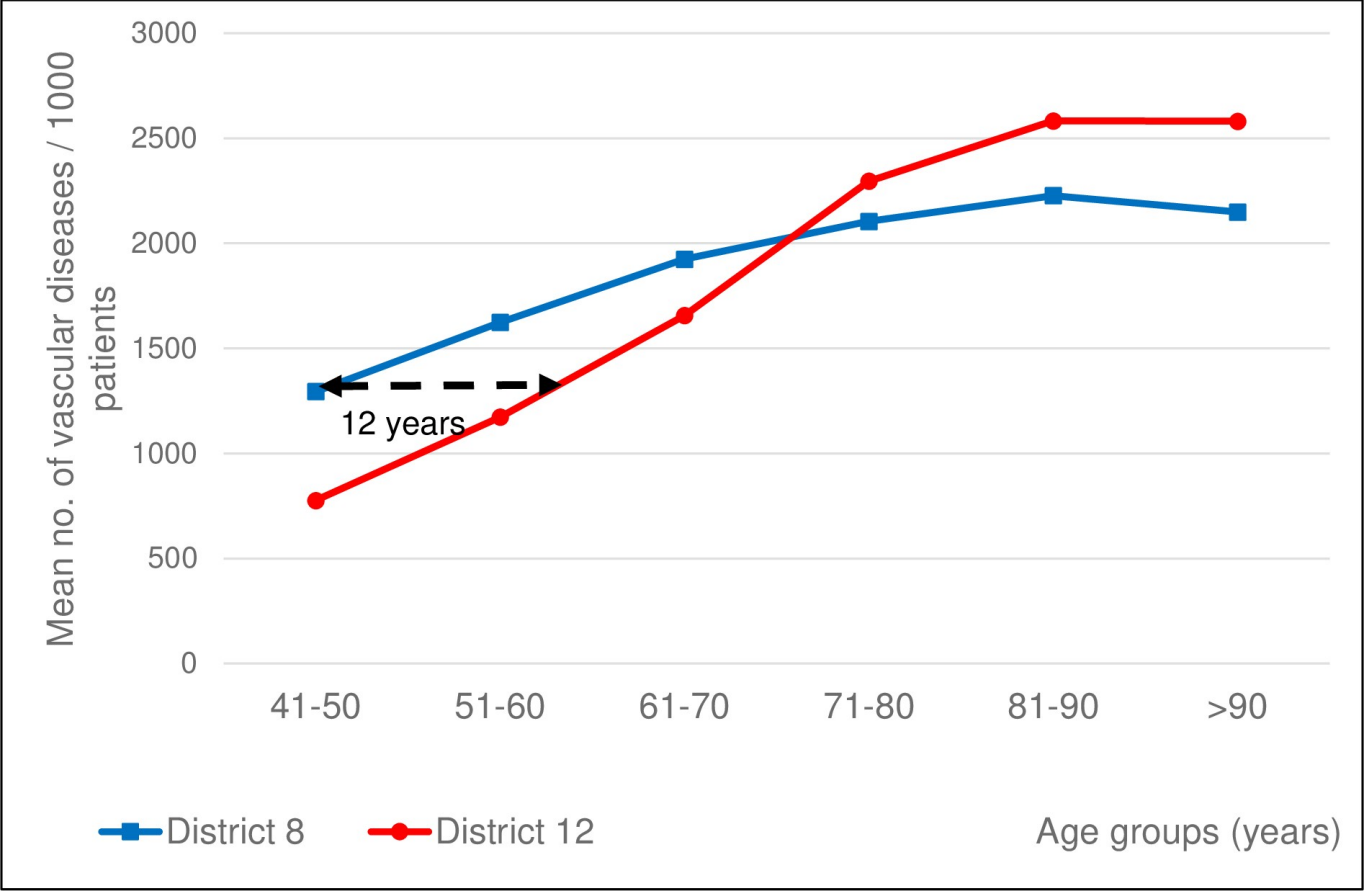
Number of associated cardiovascular diagnoses in ischemic stroke patients in 10-year age groups. Mean number of associated cardiovascular diagnoses per 1000 ischemic stroke patients of the two districts in 10-year age groups. Patients dwelling in District 8 being 41–50 years of age at the index ischemic stroke have a vascular disease burden as high as their 12 years’ older fellows from District 12 based on linear interpolation.

When taking into consideration cardiovascular diagnoses (hypertension, atrial fibrillation, arrhythmias, ischemic heart disease, congestive heart disease), we have found a similar correlation: the average number of associated cardiovascular diseases of District 8 ischemic stroke patients of the 5^th^, 6^th^ and 7^th^ decades exceeds significantly that of District 12 (Fig 9). The trendlines cross over in the age-group of the 8^th^ decade of life. In the very elderly we can notice a higher frequency of vascular diseases in District 12.

**Fig 10:**
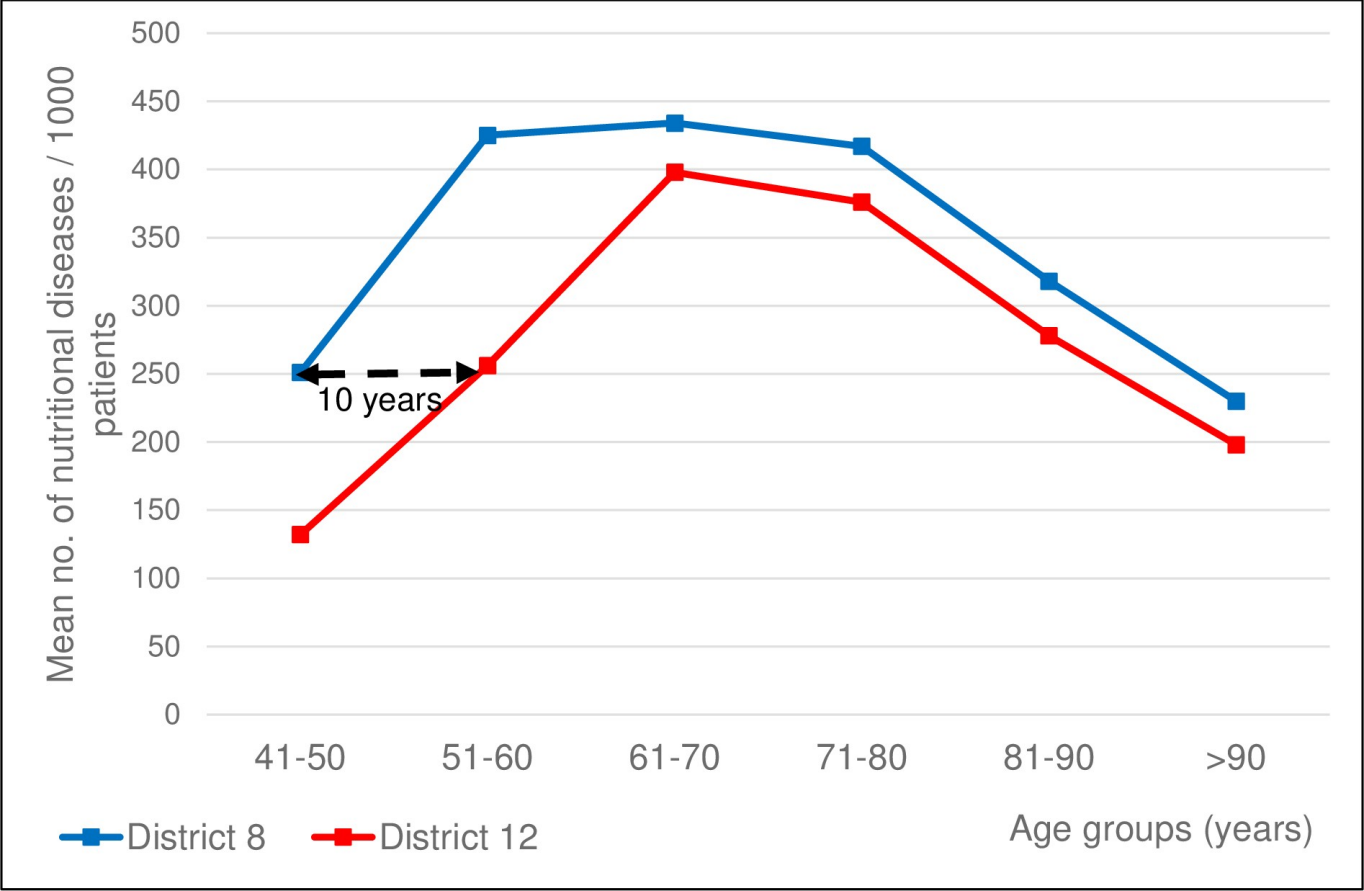
Number of associated nutritional diseases per 1000 ischemic stroke patients in 10-year age groups. Mean number of associated nutritional diseases per 1000 ischemic stroke patients of the two districts in 10-year age groups. Patients dwelling in District 8 being 41–50 years of age at ischemic stroke onset have a nutritional disease burden as high as their 10 years’ older fellows from District 12.

Considering nutritional disorders (diabetes, obesity and hyperlipidemia), we have observed that District 8 ischemic stroke patients in their forties show disease prevalence similar to District 12 patients in their fifties. The prevalence of nutritional diseases in District 8 patients is higher than that of District 12 in all age-groups (Fig 10).

The prevalence of hypertension and diabetes at index was higher in the ischemic stroke cases of District 8, while we haven’t found any difference for atrial fibrillation or hyperlipidemia (Table 4).

**Table 4:** Prevalence of hypertension, diabetes, atrial fibrillation and hyperlipidemia at the index ischemic stroke in the two neighborhoods.

	District 8	District 12	Significance
Hypertension	75%	66%	p<0.001
Diabetes	26%	16%	p<0.001
Atrial fibrillation	13%	15%	ns.
Hyperlipidemia	8%	10%	ns.

The proportion of hospital readmissions of any cause or the ratio of recurrent ACV cases of the same or of different subtype than before was not different in the two cohorts.

## Discussion

In our study comparing the most and least wealthy districts of a capital city in Central Europe we found that stroke in the less wealthy neighborhood hits at a younger age, and incidence as well as mortality is higher compared to the wealthier district. Despite the younger age of patients and consequently lower short term case fatality in the less wealthy district, case fatality converges in the long-term and by 10 years after the index event case fatality becomes similar in the two districts. Higher late case fatality of the 40–70 year-old patients in the poor neighborhood is responsible for this convergence.

### Comparison of incidence and outcome to values reported for Hungary

The incidence of ACV reported in our study is lower for both districts compared to the value reported for Hungary [20, 21], which in change is one of the highest amongst European countries [22, 23]. This raises the question whether the capital itself is affected less than the rural areas. This question is yet to be answered. Recent epidemiological data about incidence and outcome of cerebrovascular diseases in socially and economically divergent subregions of Hungary are scarce. Some suggest the capital belongs to the regions of lower stroke incidence and mortality areas of Hungary [21].

Using age-standardized data, the American Heart Association 2014 update on heart disease and stroke statistics reported stroke mortality of 109/100,000 for Hungary’s male population, the country ranking 4th on an international level [2]. Our study’s estimates considering all-cause deaths of ACV and ischemic stroke patients are higher in District 8 and somewhat lower in District 12 than the age standardized stroke mortality data cited above for internationally top-ranking Hungary. All our study’s estimates exceed the age-adjusted mortality rates reported in most European countries [2]. An example for the opposite end of the spectrum could be that shown by an epidemiologic study performed in Catalonia (Spain) [23]. This study found that the age-adjusted cerebrovascular disease mortality rates were 58/100,000 (men) and 43/100,000 (women), among the lowest in developed countries.

The differences of the two neighborhoods’ incidence, fatality and mortality reported by us are somewhat higher compared to the numbers estimated by a prior District 8–12 study [15] using different methodology, much smaller cohort and shorter follow-up.

In spite of the forecasted difficulty of demonstrating the effect of income differences in populations smaller than 800,000 inhabitants [9], we found that the visible discrepancy in income levels is indeed associated with a substantial difference of outcome even in neighborhoods with a few 10,000 residents.

In a supplementary analysis beyond the two “extreme” districts of Budapest, we tested if age at stroke onset is indeed associated with living standard. In this analysis we used annual taxable income as a surrogate for living standard, and we related income to age at stroke onset using data from all the 23 districts of Budapest. Supporting our detailed results from the two extreme districts, this supplementary analysis suggests that SES even within one city is associated with at least one feature of stroke: the lower the annual income the lower the age at stroke onset.

### Socioeconomic gap and divergence in incidence and outcome

We suppose that the gap between the poor and the wealthy noticeable on the one hand in the acute phase, and on the other hand during long-term follow-up, might be explained by different factors. Provenance from a poorer neighborhood might intervene in both phases.

Regarding the acute phase, we saw that patients of District 8 are younger at disease onset, and age-standardized incidence of ACV overall and ischemic stroke specifically, is higher. In spite of the younger age of District 8 patients, 30 days’ case fatality of ACV was similar with that of District 12 patients, while in case of ischemic stroke, acute case fatality was understandingly lower for the 5 years’ younger group of District 8 than for District 12.

Stroke features related to the acute phase (age at onset, case severity and incidence) might be influenced by the higher prevalence of the pre-stroke lifestyle risk factors and risk diseases in the poorer district. Nevertheless, some authors [12] suggest that above all these, belonging to a disadvantaged neighborhood might affect stroke characteristics directly, representing an independent risk factor. Unfortunately, our study does not add to differentiating between the direct or indirect effect of provenance as we have not studied the lifestyle risk factors.

Regarding the chronic phase, we found that the younger population of District 8 catches up in case fatality with the older population of District 12. By long-term follow-up, District 8 group exceeds that of District 12 in fatality of ACV and becomes similar with that of District 12 regarding fatality of ischemic stroke.

As the results about the fatality of ACV overall and ischemic stroke specifically of District 8 and District 12 have shown, the gap affecting the younger patients of the poorer neighborhood increases with time passing, while there is no significant difference among the elderly. It is as if the younger patients of District 8 would age faster under the burden of the comorbidities even prior to the stroke and as if this process would speed up after being hit by the stroke.

The difference between the poor and the wealthy becomes ampler with time passing from the acute event, expressed in higher long-term fatality. This long-time effect might have a three-fold explanation: there could be differences on individual level (e.g. adherence to secondary prevention, changing lifestyle risk factors), in the coping mechanisms of the families of patients (social and financial resources might influence home long-term care) and on institutional level (e.g. differences in availability of in-hospital rehabilitation or chronic long-term facilities). Although there are some governmental programs targeting secondary prevention, not much attention is given to offer home-based and institutional long-term care more equitable among stroke populations of different SES.

### Comorbidities

We consider the compellingly more comorbidities, the higher frequency of vascular and nutritional diseases associated to the ischemic stroke cases of District 8 as a possible explanation of the higher incidence and mortality of ischemic stroke found in District 8 compared to District 12. The prevalence of hypertension in ischemic stroke (75% and 66% in District 8 and 12) is comparable to the numbers reported in other Hungarian stroke cohorts: 81% by Bereczki et al [24] and 75% by Aszalos and colleagues [25]. All these studies, including ours, have found a considerably higher prevalence of hypertension compared to the one reported by the European survey: approximately 50% [26]. Diabetes was more prevalent in the disadvantaged district (26% vs 16% in District 12), and even more prevalent than formerly reported in Eastern Hungary [24,27].

Albeit the universal healthcare system offers a potential for uniform quality of disease management, and this could mean that the more prevalent risk diseases of District 8 could be controlled with more resources, our data showing higher ACV and ischemic stroke incidence and worse outcome in this neighborhood’s working-age inhabitants suggest that this isn’t the case.

### Validity of database

Optimally we would have identified the patients in a prospective manner, with individual case certification. Nevertheless, based on a recent report by Ajtay et al, databases created from hospital reports submitted for reimbursement purposes to the National Health Insurance Fund can be used reliably in Hungary for epidemiological studies on cerebral infarction. They have demonstrated that the diagnoses reported to the health insurance fund correspond to those given to the patients in their discharge reports in 99% of cases [28]. We excluded those patients hospitalized in the previous 2 years for ischemic stroke or intracerebral hemorrhage in order to rule out those cases from the study that might represent the chronic cases after a stroke. Thus we are confident that our study represents a reliable approximation of acute case cohorts.

### Limitations of our study

Our study has several limitations. First, verifying the accuracy of hospital discharge codes was not possible due to the design of the study. That would have needed a personal individual case certification, which is incompatible with epidemiological studies based on anonymized administrative data.

Second, although we found higher prevalence and earlier appearance of some stroke risk diseases, we could not identify all intermediating factors between SES and disease outcome. The association of living standard with stroke outcome is well documented, involving several factors like level of education, personal income etc. all needing individual evaluation [3,7]. Folyovich et al have found in the same two districts that smoking, alcohol consumption and poorly controlled hypertension were more prevalent in the poorer neighborhood [15]. These data are not accessible in epidemiological studies like ours based on anonymized registers. Still, neighborhood income has already been used as an acceptable substitute for individual SES in several studies [29,30].

Third, we lack data regarding the level of control of risk diseases. Although we found higher prevalence and earlier appearance of some risk diseases, we have no data regarding the adherence to measures of primary and secondary prevention in the study population.

Fourth, we could consider only cases admitted to hospital as representing all incident cases. By this we probably underestimate the incidence of ACV and ischemic stroke, as our numbers do not include those not admitted to hospital. Hospitalization rate of stroke cases was reported to be of 84–95% in different European registers [31–33].

Fifth, we do not have access to individual patient records, therefore we lack data regarding case severity at onset for the studied cohorts. Besides the already demonstrated higher prevalence of comorbidities, stroke severity is a well-known predictor of case fatality [34,35]. Having in view our data of younger ACV patients of District 8 showing similar acute fatality as the older group of District 12, one might suppose that this might also be explained through worse case severity of District 8 cases. We also lack data regarding the incidence and mortality of the different subtypes of ischemic stroke according to the TOAST criteria, which was a significant predictor for long-term survival [36]. Future studies should analyze data both according to subtypes and acute case severity in the study population.

Finally, as this study was a retrospective observational cohort study based on the database of the National Health Insurance Fund, we do not have the exact cause of death. We know only the date of deaths of any cause for patients hospitalized initially for ACV from both districts. The fatality of the first year after the disease onset is probably closer associated to the fatality attributable to the disease itself than any fatality noticed later on. Still, we consider that the fatality observed during the 5–12 years’ follow-up period could further contribute to understanding the gap between the two neighborhoods.

Our study has several **strengths** as well. First, we could analyze a consistent data source, covering all subjects residing in the 2 districts of the city. Second, the 6-year long inclusion period provided a sufficient number of patients for analysis, including the option to study age groups within the cohorts. Third, data linkage of the anonymized patient records made it possible for us to obtain information on risk diseases. Finally, data were available for an over 5-year-long follow-up for all patients, thus long term prognosis of ACV could be evaluated and compared between districts with extreme socioeconomic conditions within one city.

As far as we know, no similar comprehensive analysis was performed in any Central-Eastern European population of this size. We intend to continue this study including patients with strokes of all types, during 10 years, performing a long-term follow-up focusing on any diverging trend between acute and long-term case fatality and on finding the probable explanation.

## Conclusions

Despite the availability of a universal healthcare system, in the poorer neighborhood of one single city stroke hits significantly earlier and harder. The impact is most evident in the working-age groups. In the younger of the poorer district the incidence of ACV and ischemic stroke, and the number of comorbidities are higher, death occurs more frequently and survival after onset is shorter. More prevalent hypertension and diabetes, and the over 10 years earlier appearance of cardiovascular and metabolic comorbidities may partly explain the younger age at ACV onset and the worse long term prognosis in the disadvantaged district. Insufficient use of institutional rehabilitation, the quality of home care and the lack of adherence to measures of secondary prevention may also add to the worse outcome in the less wealthy region. Identifying a vulnerable subgroup of the society obliges us to apply more intensive prevention strategies in this target group.

After having seen these differences in both incidence and mortality between the poorer and wealthier districts, we consider it a challenge to detect and study in-depth the possible link between the poverty and the fate of stroke patients diverging within this hypothetically universal healthcare system.

## Supporting information

S1 Table Original database of the study population (Szocs et al database.xlsx). (XLSX)
